# Bioinformatic analysis identifies potential key genes of epilepsy

**DOI:** 10.1371/journal.pone.0254326

**Published:** 2021-09-23

**Authors:** Yike Zhu, Dan Huang, Zhongyan Zhao, Chuansen Lu

**Affiliations:** 1 Department of Respiratory Medicine, Hainan General Hospital, Haikou, China; 2 Department of Neurology, Hainan General Hospital, Haikou, China; Fu Jen Catholic University, TAIWAN

## Abstract

**Background:**

Epilepsy is one of the most common brain disorders worldwide. It is usually hard to be identified properly, and a third of patients are drug-resistant. Genes related to the progression and prognosis of epilepsy are particularly needed to be identified.

**Methods:**

In our study, we downloaded the Gene Expression Omnibus (GEO) microarray expression profiling dataset GSE143272. Differentially expressed genes (DEGs) with a fold change (FC) >1.2 and a P-value <0.05 were identified by GEO2R and grouped in male, female and overlapping DEGs. Functional enrichment analysis and Protein-Protein Interaction (PPI) network analysis were performed.

**Results:**

In total, 183 DEGs overlapped (77 ups and 106 downs), 302 DEGs (185 ups and 117 downs) in the male dataset, and 750 DEGs (464 ups and 286 downs) in the female dataset were obtained from the GSE143272 dataset. These DEGs were markedly enriched under various Gene Ontology (GO) terms and Kyoto Encyclopedia of Genes and Genomes (KEGG) terms. 16 following hub genes were identified based on PPI network analysis: ADCY7, C3AR1, DEGS1, CXCL1 in male-specific DEGs, TOLLIP, ORM1, ELANE, QPCT in female-specific DEGs and FCAR, CD3G, CLEC12A, MOSPD2, CD3D, ALDH3B1, GPR97, PLAUR in overlapping DEGs.

**Conclusion:**

This discovery-driven study may be useful to provide a novel insight into the diagnosis and treatment of epilepsy. However, more experiments are needed in the future to study the functional roles of these genes in epilepsy.

## 1 Introduction

Epilepsy, one of the most common brain conditions including both genetic and acquired disorders, affects at least 46 million people worldwide [1]. As a complex diagnosis consisting of multiple subtypes, it is usually hard to be identified properly. People with epilepsy have varied symptoms such as strange sensations, emotions, and behavior or convulsions, muscle spasms, and loss of consciousness when the brain sends out the wrong signals. Antiepileptic drugs are the main treatment and increasing nowadays. However, there are still up to a third of people who have drug-resistant epilepsy [2, 3]. Gender differences in epilepsy are observed in clinical and experimental researches [4]. It is suggested that the incidence of epilepsy is slightly lower in females than in males, and males have greater mortality [5]. Several studies show that unprovoked seizures and status epilepticus are more common in males as compared with females [6–9], whereas some idiopathic generalized epilepsies are more frequent in females [10–12].

Despite a number of genes and signaling pathways in the development and progression of epilepsy have been widely studied, the mechanisms underlying epilepsy are still being unraveled. Currently, the high-throughput sequencing analysis of gene expression, coupled with bioinformatics tools, becomes promising for investigating the novel genes in the initiation and evolution of diseases. In the study, we researched the human peripheral blood sample microarray dataset GSE143272 from the GEO to identify the DEGs between epilepsy patients and normal individuals by applying the bioinformatic method. Our results may provide potential biomarker candidates for clinical diagnosis and therapy of epilepsy.

## 2 Materials and methods

### 2.1 Microarray data

We downloaded the microarray expression profiling dataset GSE143272 as peripheral blood expression profiles of patients with epilepsy, deposited by Rawat C et al., from the GEO (https://www.ncbi.nlm.nih.gov/geo/). The dataset was performed on GPL10558 Illumina HumanHT-12 V4.0 expression beadchip platform. The array data for GSE143272 contained 142 samples, including 34 newly diagnosed, drug-free patients with epilepsy, 57 followed-up patients receiving antiepileptic drug monotherapy, and 50 healthy subjects. We selected the drug-free epilepsy patients and healthy control subjects, consisting of 21 male epilepsy patients, 13 female epilepsy patients, 26 male controls, and 25 female controls. All data were downloaded from the GEO freely. No ethics approval and patients’ informed consent were needed for this present study.

### 2.2 Identification of DEGs

The online analysis tool GEO2R was using to identify the DEGs. Genes with the specific cut-off criteria of FC >1.2 and a P-value <0.05 were considered DEGs. The epilepsy patients and healthy controls were assigned to two groups depending on gender, male and female, according to the annotation of the GSE143272. We conducted the analysis by comparing the male epilepsy patients with male controls, and female epilepsy patients with female controls, respectively. The intersecting and sex-specific genes were obtained by drawing a Venn diagram with Bio-Conductor R and package. The visual hierarchical cluster analysis was also performed, exhibiting a volcano plot of DEGs.

### 2.3 Functional enrichment analysis of DEGs

We used the online database webgestalt (http://www.webgestalt.org/) to reveal the functions of DEGs, by conducting the analysis including GO annotation and KEGG pathway enrichment analyses. The GO analysis consisted of three divisions: biological process (BP), cellular component (CC), and molecular function (MF). P <0.05 and an enriched gene count ≥5 were selected as the criteria for statistical significance.

### 2.4 Protein-Protein Interaction (PPI) network analysis

We applied the online database STRING (https://string-db.org/) to perform the PPI network analysis of evaluating the protein-protein interactions between the screened DEGs. The STRING, a PPI database, collects and assesses evidence from many sources, such as scientific literature, to integrate all known and predicted associations between proteins, including physical interactions and functional associations [13]. All PPI pairs with an interaction score of >0.9 as the threshold value were extracted. Moreover, we used Cytoscape v3.7.2 software plugin cytoHubba to calculate the degree of all nodes and construct PPI networks through different topological analyses. Cytoscape, an open source software project that integrates biomolecular interaction networks with high-throughput expression data and other molecular states into a unified conceptual framework, is one of the most powerful network biology analyses and visualization tools when applied in conjunction with large databases of protein-protein, protein-DNA, and genetic interactions [14, 15]. CytoHubba was composed by Chia-Hao Chin et al. for ranking and exploring important nodes in biological networks by their network features, which provides 11 topological analysis methods in one-stop shopping way [16]. The proteins with higher degrees are more likely to be essential proteins, and the importance of nodes within a biological network will be evaluated. These node ranking methods can be divided into two categories: local and global methods. The local-based methods are considered to be the better methods in discovering essential proteins, which only focus on the relationship between the node and its direct neighbors. Due to the heterogeneity of biological networks, it is reasonable to implement multiple methods for capturing essential proteins [16]. Thus, the genes with the top 10 highest degree values were screened by the 3 topological analyses, including maximal clique centrality (MCC), maximum neighborhood component (MNC), and Degree, all of which are local-based methods. The overlapping hub genes in the top 10 by these 3 topological methods were identified.

## 3 Results

### 3.1 Identification of DEGs

**Fig 1:**
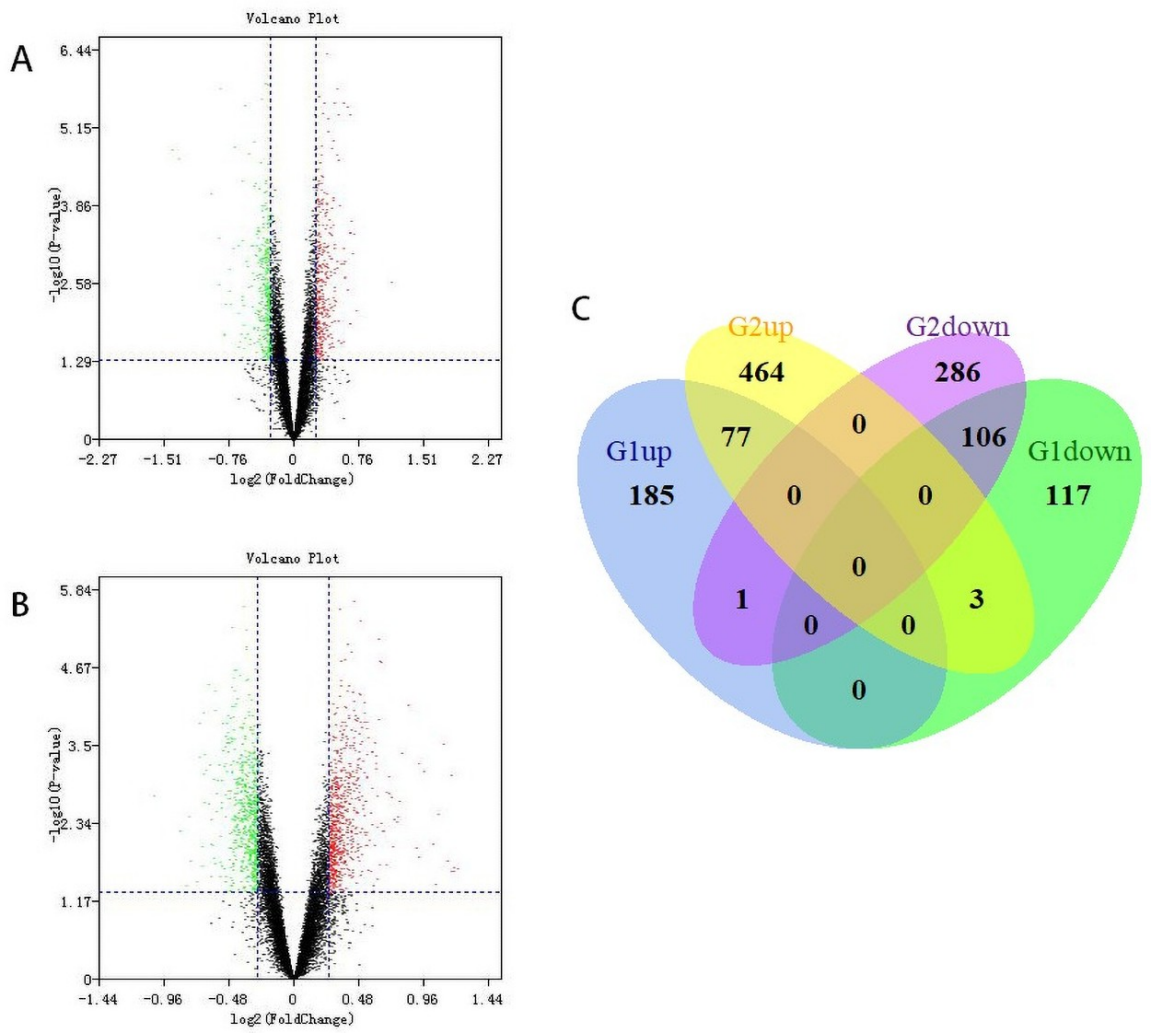
Identification of DEGs from GSE143272. (A) Volcano plot of DEGs in the male dataset. (B) Volcano plot of DEGs in the female dataset. Red, upregulation; green, downregulation. (C) Venn diagram of upregulated and downregulated DEGs based on the male (G1) and female (G2) datasets.

We downloaded the dataset GSE143272 from the GEO database, using GEO2R to analyze the DEGs between drug-free epilepsy patients and normal individuals. Epilepsy patients consisted of idiopathic, cryptogenic, and symptomatic epilepsy. In total, 263 upregulated and 226 downregulated DEGs were identified between male epilepsy patients and male controls, while 544 upregulated and 393 downregulated DEGs were identified between female epilepsy patients and female controls. By conducting Venn analysis, we examined the overlap among the two datasets and obtained a total of 183 (77 upregulated and 106 downregulated) DEGs. Besides, Sex-specific DEGs were screened, within which 302 (185 upregulated and 117 downregulated) DEGs were obtained in the male dataset, while 750 (464 upregulated and 286 downregulated) DEGs were obtained in the female dataset. The volcano plot and Venn diagram for the DEGs are presented in Fig 1.

### 3.2 Functional enrichment analysis of DEGs

**Fig 2:**
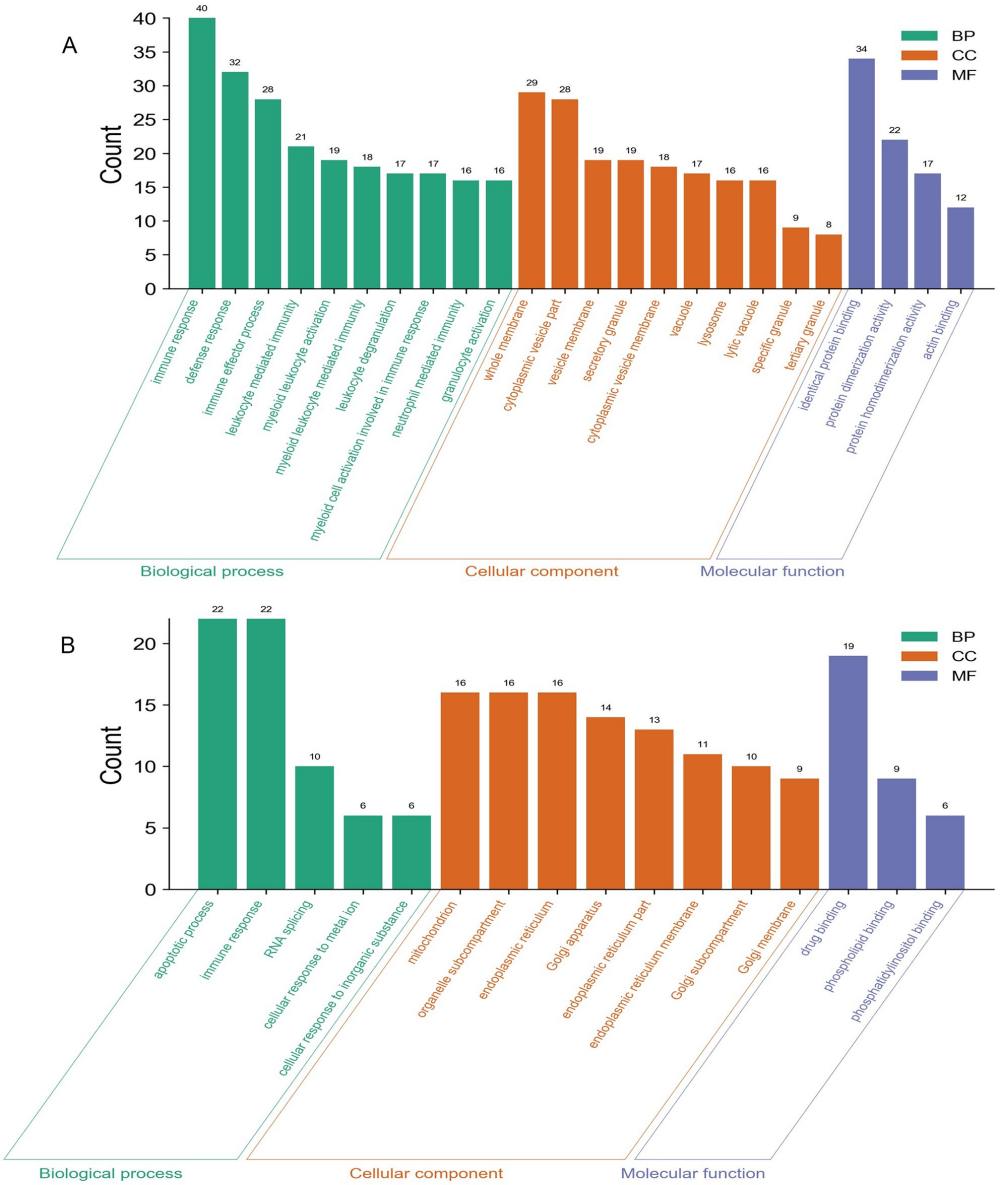
GO analyses of the male-specific upregulated (A) and downregulated (B). The y-axis depicts the number of genes. The x-axis lists the enriched functional terms. The color of bars corresponds to different categories of GO analysis (green represents BP, orange represents CC, and purple represents MF).

**Fig 3:**
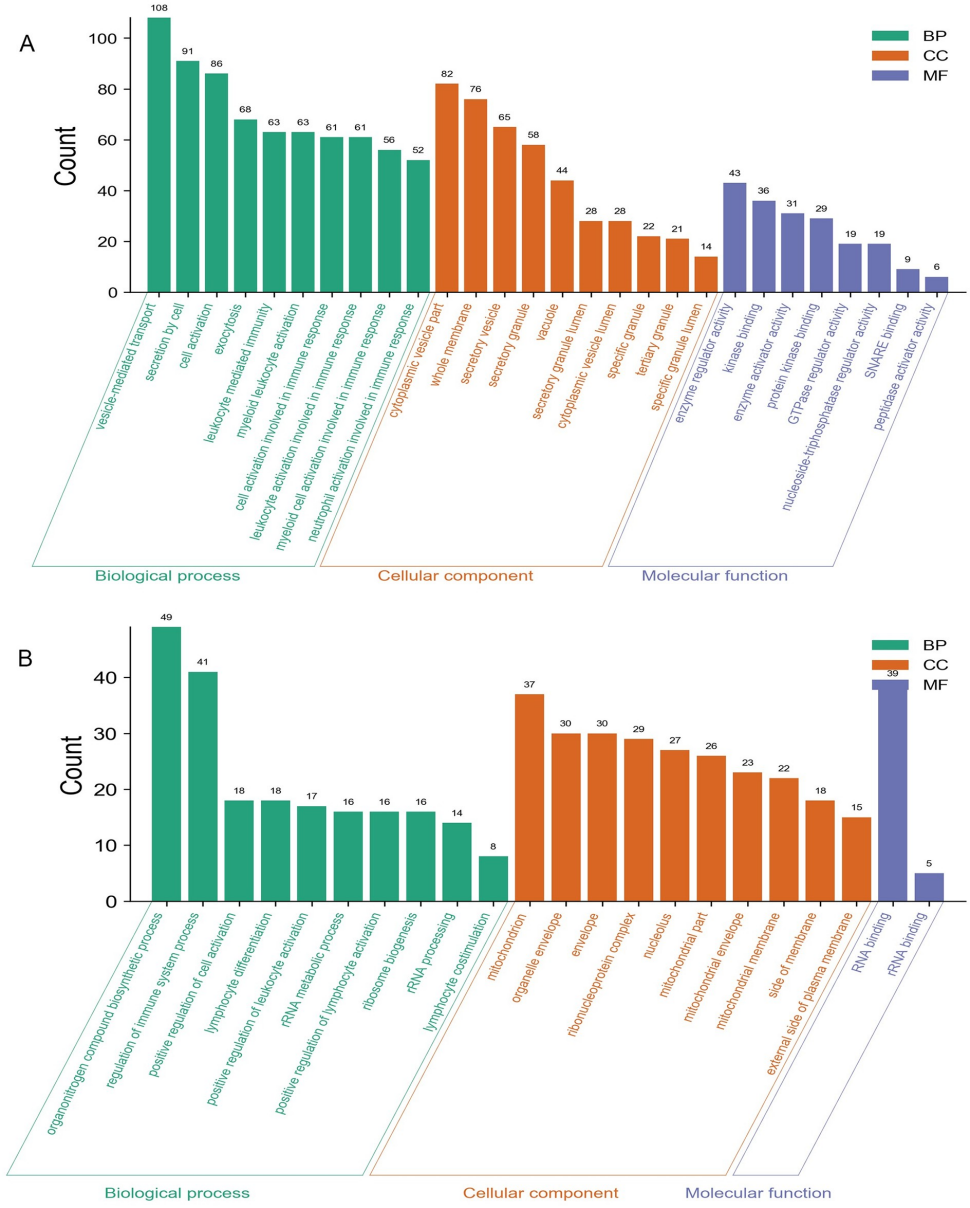
GO analyses of the female-specific upregulated (A) and downregulated (B). The y-axis depicts the number of genes. The x-axis lists the enriched functional terms. The color of bars corresponds to different categories of GO analysis (green represents BP, orange represents CC, and purple represents MF).

**Fig 4:**
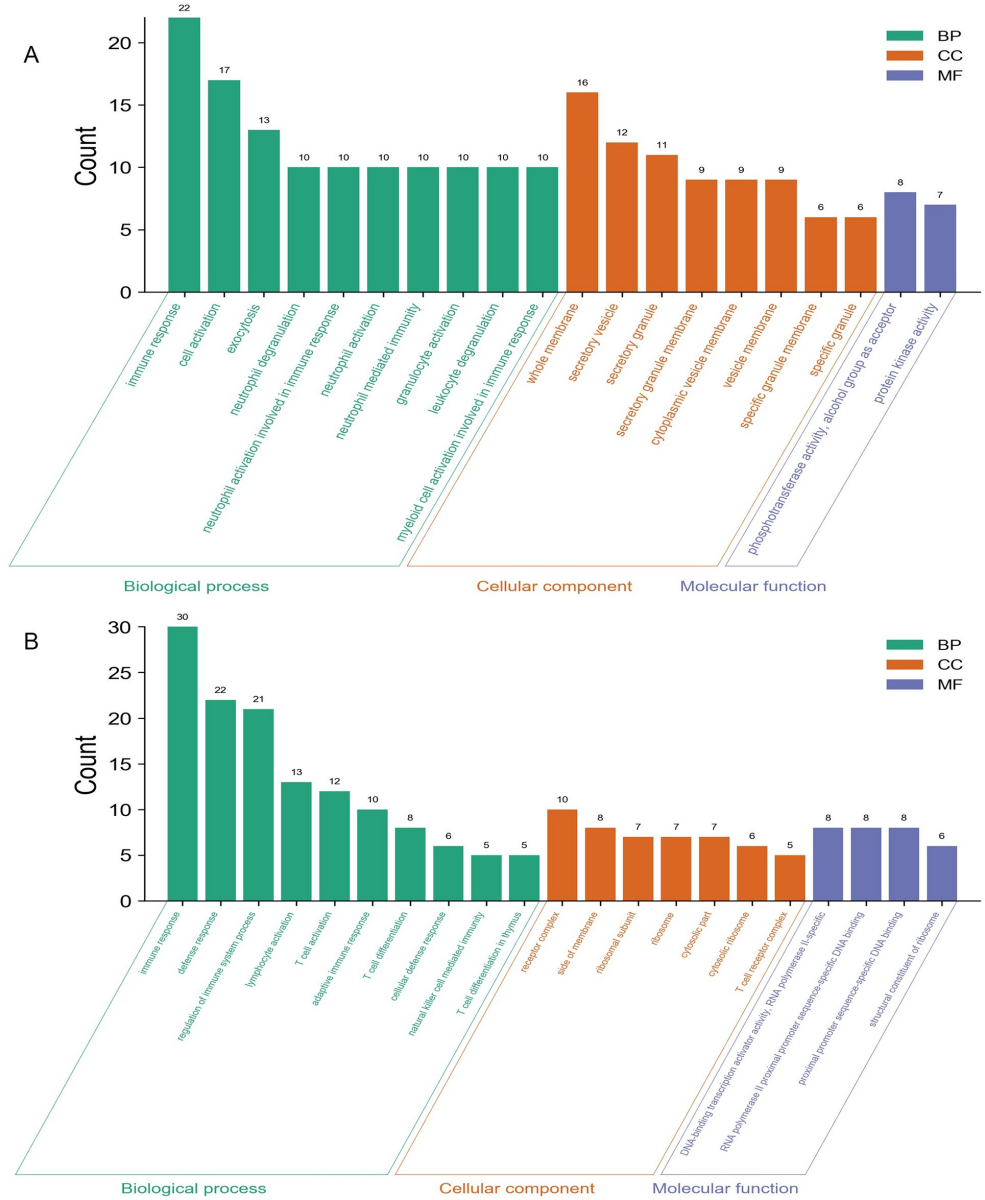
GO analyses of the overlapping upregulated (A) and downregulated (B) DEGs. The y-axis depicts the number of genes. The x-axis lists the enriched functional terms. The color of bars corresponds to different categories of GO analysis (green represents BP, orange represents CC, and purple represents MF).

We carried out the functional enrichment analysis for the male-specific DEGs, female-specific DEGs, and overlapping DEGs. The upregulated and downregulated DEGs were analyzed respectively. Top enriched GO terms were shown in Figs 2–4. Results indicated that the male-specific upregulated DEGs were mostly enriched related to immune response in BP, cytoplasmic vesicle part in CC, and identical protein binding in MF term, while male-specific downregulated DEGs were mostly enriched related to RNA splicing in BP, mitochondrion in CC, and phospholipid binding in MF term; the female-specific upregulated DEGs were mostly enriched related to vesicle-mediated transport in BP, cytoplasmic vesicle part in CC, and enzyme activator activity in MF term, while female-specific downregulated DEGs were mostly enriched related to rRNA metabolic process in BP, ribonucleoprotein complex in CC, and RNA binding in MF term; the overlapping upregulated DEGs were mostly enriched in immune response for BP, secretory granule membrane for CC, and phosphotransferase activity, alcohol group as acceptor for MF term, while overlapping downregulated DEGs were mostly enriched in immune response for BP, T cell receptor complex for CC, and structural constituent of ribosome for MF term, respectively.

In addition, the top KEGG pathways were presented in Table 1. The male-specific upregulated and downregulated, female-specific upregulated and downregulated, overlapping upregulated and downregulated DEGs were mostly significantly enriched in sphingolipid signaling pathway, rheumatoid arthritis, leishmaniasis, natural killer cell mediated cytotoxicity, platelet activation, and structural constituent of ribosome, respectively.

**Table 1:** KEGG pathway enrichment analyses for DEGs.

Gender		Pathway description	P-Value	Count
Male	Upregulated	Sphingolipid signaling pathway	0.000462	7
		Biosynthesis of amino acids	0.001856	5
		Hepatocellular carcinoma	0.003485	7
		Autophagy	0.003942	6
		NOD-like receptor signaling pathway	0.014277	6
	Downregulated	Rheumatoid arthritis	0.000121	5
		Chemokine signaling pathway	0.003538	5
		Human cytomegalovirus infection	0.007385	5
		Cytokine-cytokine receptor interaction	0.021566	5
Female	Upregulated	Leishmaniasis	0.000008	11
		Osteoclast differentiation	0.000076	13
		Chemokine signaling pathway	0.000105	16
		Renal cell carcinoma	0.000148	9
		Hepatitis B	0.000252	13
		Systemic lupus erythematosus	0.000437	12
		Kaposi sarcoma-associated herpesvirus infection	0.000937	14
		Autophagy	0.001133	11
		Human immunodeficiency virus 1 infection	0.001169	15
		Phagosome	0.001433	12
	Downregulated	Natural killer cell mediated cytotoxicity	3.85E-07	13
		Antigen processing and presentation	0.000366	7
		Graft-versus-host disease	0.000680	5
		Th1 and Th2 cell differentiation	0.001076	7
		NF-kappa B signaling pathway	0.001300	7
		T cell receptor signaling pathway	0.001856	7
		Sphingolipid signaling pathway	0.004458	7
		Hematopoietic cell lineage	0.006826	6
		HIF-1 signaling pathway	0.007894	6
Overlap	Upregulated	Platelet activation	0.000410	5
	Downregulated	Structural constituent of ribosome	0.000097	6
		DNA-binding transcription activator activity, RNA polymerase II-specific	0.001188	8
		RNA polymerase II proximal promoter sequence-specific DNA binding	0.002694	8
		proximal promoter sequence-specific DNA binding	0.003296	8

### 3.3 PPI network analysis of DEGs

**Fig 5:**
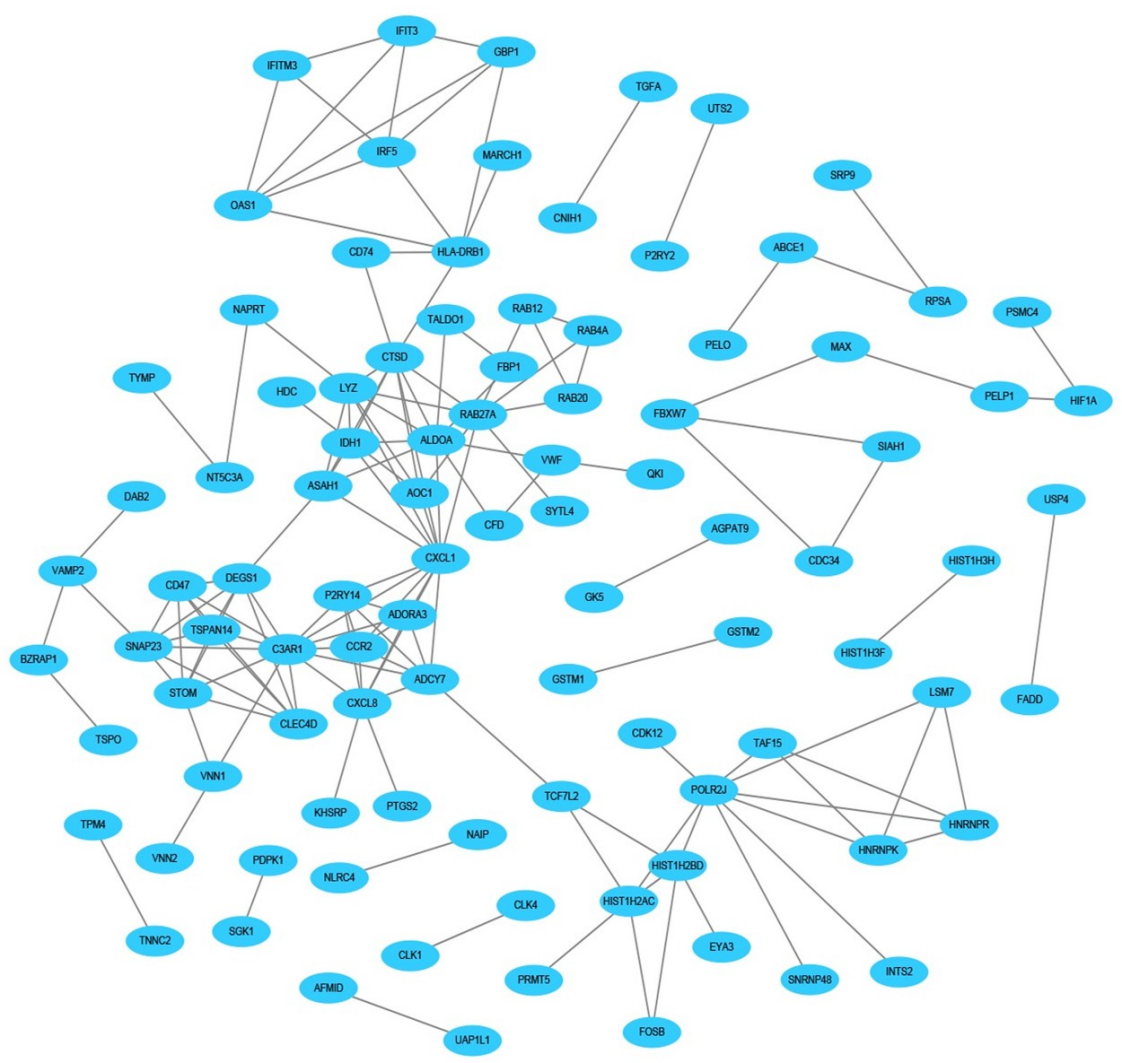
Male-specific PPI network in epilepsy. The PPI network included 96 nodes and 151 edges.

**Fig 6:**
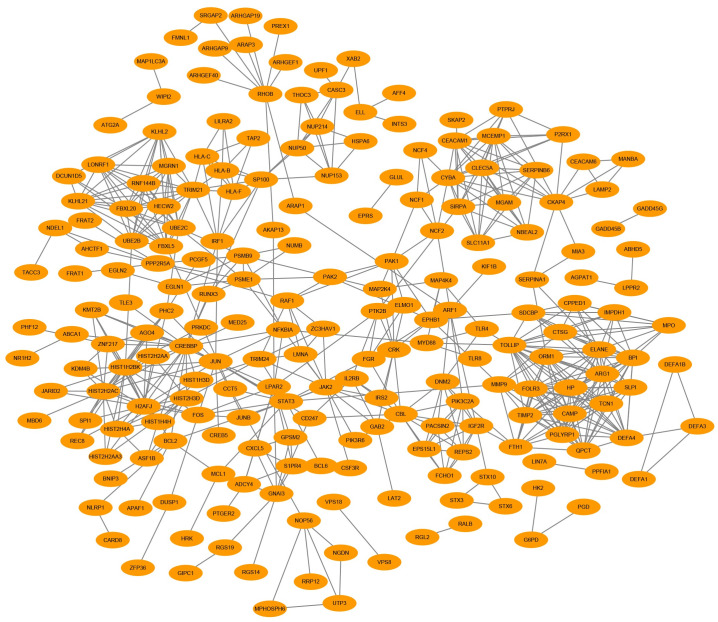
Female-specific PPI network in epilepsy. The PPI network included 213 nodes and 604 edges.

**Fig 7:**
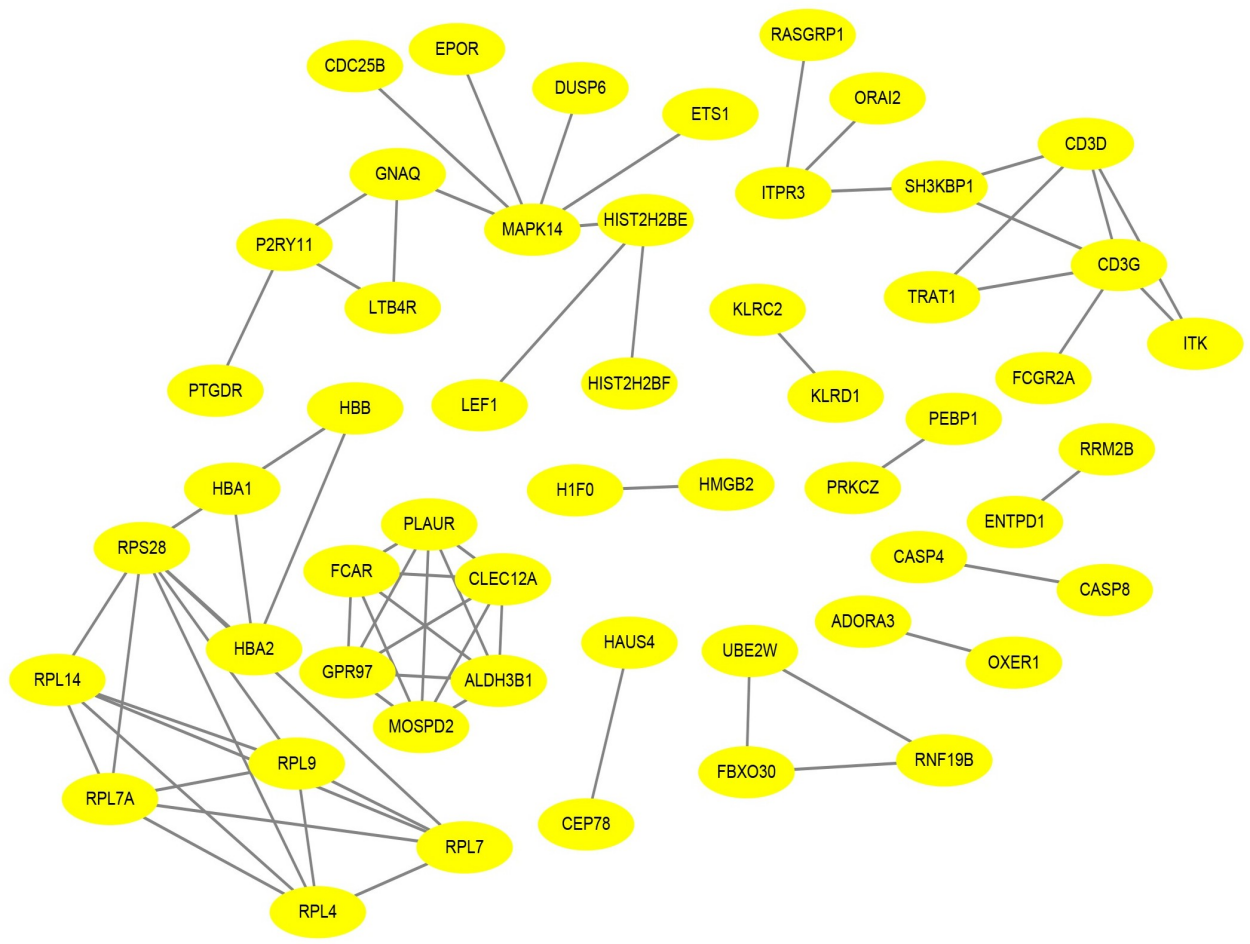
Male and female overlapping PPI network in epilepsy. The PPI network included 53 nodes and 68 edges.

The STRING database was applied to determine the PPI networks with interaction score > 0.9 among the male-specific, female-specific, and overlapping DEGs, which were constructed via Cytoscape software, respectively (Figs 5–7).

Cytohubba plugin of Cytoscape was used to rank the top 10 nodes in the above PPI networks according to 3 topological analysis methods, including MCC, MNC, and Degree. The overlapping hub genes according to the three methods were ADCY7, C3AR1, DEGS1, CXCL1 in male-specific DEGs, TOLLIP, ORM1, ELANE, QPCT in female-specific DEGs, and FCAR, CD3G, CLEC12A, MOSPD2, CD3D, ALDH3B1, GPR97, PLAUR in overlapping DEGs (Table 2).

**Table 2:** Hub genes for highly differentiated expressed genes ranked in Cytohubba plugin of Cytoscape.

Gender	Rank methods in CytoHubba		
Male	MCC	MNC	Degree
	***ADCY7***	***ADCY7***	***ADCY7***
	ADORA3	ADORA3	***C3AR1***
	P2RY14	P2RY14	LYZ
	***C3AR1***	***C3AR1***	POLR2J
	STOM	LYZ	***DEGS1***
	CLEC4D	STOM	RAB27A
	SNAP23	CLEC4D	ALDOA
	***DEGS1***	***DEGS1***	***CXCL1***
	***CXCL1***	***CXCL1***	CTSD
	CXCL8	CTSD	CXCL8
Female	***TOLLIP***	***TOLLIP***	***TOLLIP***
	PGLYRP1	ARG1	ARG1
	***ORM1***	CREBBP	CREBBP
	TCN1	***ORM1***	***ORM1***
	CAMP	BPI	BPI
	TIMP2	H2AFJ	H2AFJ
	FOLR3	HIST1H2BK	HIST1H2BK
	HP	HIST2H2AC	HIST2H2AC
	***ELANE***	***ELANE***	***ELANE***
	***QPCT***	***QPCT***	***QPCT***
Overlap	***FCAR***	***FCAR***	***FCAR***
	***CD3G***	***CD3G***	***CD3G***
	***CLEC12A***	***CLEC12A***	***CLEC12A***
	***MOSPD2***	***MOSPD2***	GNAQ
	MAPK14	***CD3D***	***MOSPD2***
	***CD3D***	***ALDH3B1***	MAPK14
	***ALDH3B1***	***GPR97***	***CD3D***
	***GPR97***	HBA1	***ALDH3B1***
	HBA1	***PLAUR***	***GPR97***
	***PLAUR***	HBA2	***PLAUR***

## 4 Discussion

In this study, we performed bioinformatics analysis to search for the potential key genes associated with epilepsy. Male and female epilepsy were compared to healthy controls respectively on the hypothesis that epilepsy in different genders had different mechanisms. The results showed that 302 male-specific DEGs, 750 female-specific DEGs, and 183 overlapping DEGs were successfully identified. Those DEGs were put into multi-step bioinformatic functional annotations, including GO, KEGG, and PPI analysis.

We observed that male, female and overlapping DEGs took part in similar and overlapping biological processes significantly. Most of the biological processes were involved in inflammation and immune system defense response, for example, immune response, leukocyte mediated immunity, T cell activation, etc. In KEGG analysis, sphingolipid signaling pathway was showed upregulated in male patients but downregulated in female patients. It is well known that sphingolipids, such as ceramide, sphingosine, sphingosine-1-phosphate, sphingomyelin, and gangliosides, play an important role in the regulation of steroidogenesis [17]. The sex hormones, such as androgens, estrogens, and progestogens, are thought to influence sex differences in epilepsy [18, 19]. For example, it is suggested that progesterone has anticonvulsant effects, while estradiol has neuroprotective effects along with mild anticonvulsant effects [4]; and androgen can amplify sex differences in the expression of some epileptic disorders [18]. The observation in our study indicated that sphingolipid signaling pathway may have a complex relationship with epilepsy in sex differences.

Based on the PPI networks, 16 hub genes were identified, which were ADCY7, C3AR1, DEGS1, CXCL1 in male-specific DEGs, TOLLIP, ORM1, ELANE, QPCT in female-specific DEGs and FCAR, CD3G, CLEC12A, MOSPD2, CD3D, ALDH3B1, GPR97, PLAUR in overlapping DEGs, respectively.

ADCY7 encodes a membrane-bound adenylate cyclase (AC), which is one of the most ubiquitous signal transduction molecules that catalyzes the formation of cyclic adenosine monophosphate (AMP) from adenosine triphosphate (ATP) [20, 21]. It is indicated that in the central amygdala (CeA) AC7 plays an important role in the modulation of presynaptic gamma-aminobutyric acid (GABA) release. When responding to ethanol and corticotropin-releasing factor (CRF), AC7 increases cAMP signaling and activates protein kinase A cascade, resulting in the release of presynaptic vesicular GABA [22]. GABA, formed within GABAergic axon terminals and released into the synapse, acts on receptors GABAA and GABAB. As the main inhibitory neurotransmitter in the cerebral cortex and hippocampus, GABA maintains inhibitory tension to balance nerve excitation. When the balance is disrupted, seizures may occur. Therefore, GABA agonists suppress seizures, while GABA antagonists cause seizures [23]. As the localization of AC7 was observed in the hippocampus, cerebral cortex, cerebellum, caudate-putamen, and nucleus accumbens [24], ADCY7 involving in regulation of GABA may be correlated with epilepsy.

C3AR1 is a Protein Coding gene. The protein C3a Receptor 1 encoded by this gene is an orphan G protein-coupled receptor for C3a, which is a proinflammatory mediator released during activation of the complement system [25]. The complement pathway is critical in innate immunity [26]. In Alzheimer’s patients, the overexpression of C3 and C3aR1 increases with cognitive decline and Braak staging. It has been shown that loss of C3aR1 in mice causes the rescue of tau pathology and attenuation of neuroinflammation, synaptic deficits, and neurodegeneration. C3aR1 is a critical regulator in neuronal tau pathogenesis and mediating central nervous system (CNS) immune network, as its direct target is STAT3 (signal transducers and activators of transcription) [27]. More and more evidence reveals there are common underlying mechanism mechanisms associated with network hyperexcitability and cognitive decline [28, 29]. Studies have shown that in mouse models the increased abnormal tau and amyloid-β proteins may have a synergistic effect on the occurrence of epileptic seizures [30–32]. Hyperphosphorylation of Tau has been reported in epileptic patients with different forms [33–37] and in a range of animal models of epilepsy [38–40]. Focus on C3AR1 mediating tau pathology might represent a novel opportunity to research therapy for epilepsy.

DEGS1 encodes Delta 4-Desaturase, Sphingolipid 1, a member of the membrane fatty acid desaturase family which is responsible for inserting double bonds into specific positions in fatty acids. The related pathways of DEGS1 are sphingolipid signaling pathway and sphingolipid metabolism [41, 42]. In the nervous system, sphingolipids are pivotal constituents of myelin formation in glial cells, which can improve the efficiency and speed of action potentials. Perturbations of the sphingolipid metabolism can result in rearrangements in the plasma membrane, which has been associated with the development of various neurological disorders [43–46]. It has been reported that a variant in DEGS1 leads to a novel early-onset autosomal recessive complex neurological disease with Intelligent disability, progressive spastic paraplegia, scoliosis, and epilepsy. The DEGS1 variant encodes C4-dihydroceramide desaturase, which plays an important role in a pathway of ceramide/phospholipids synthesis [41, 47]. In this study, the present findings also revealed that DEGS1 was correlated with epilepsy.

CXCL1 gene encodes a member of the CXC subfamily of chemokines. The CXCL1 protein plays a pivotal role by recruiting and activating neutrophils in inflammation when signaling through the CXCR2 receptor [48]. It is being increasingly recognized that immunity and inflammatory processes in the brain contribute to the pathogenesis of epilepsy [49–51]. The study reported that CXCL1 concentrations increase significantly after seizure onset, which results in a strong induction of chemotactic response from brain cells that recruits circulating neutrophils to the injured brain tissues [52]. The activated neutrophils can exacerbate the initial injury by the damage to the surrounding healthy area [53]. Thus, CXCL1 may be a novel therapeutic target for epilepsy.

TOLLIP encodes the Toll-interacting protein (Tollip), which is present in a complex with the interleukin-1 receptor associated kinase (IRAK). With the activation of IL-1β, the Tollip–IRAK complex is recruited and disrupted [54]. The overexpression of Tollip can impair IL-1β-induced activation of NF-κB, indicating that Tollip is an inhibitory modulator in inflammatory signaling [54, 55]. The cerebral cortex may display the highest density of Tollip protein [56]. Therefore, Tollip may be a potential target to provide neuroprotective effects by reducing neuroinflammation in epilepsy [57].

ORM1, accounting for 75% of plasma ORM, is an inflammatory factor with multiple activities [58]. Plasma concentration of ORM increases under the control of various regulatory mediators, including inflammatory stimuli, such as glucocorticoids, tumor necrosis factor (TNF)-α, interleukin (IL)-1, IL-8, IL-11, IL-6, and IL-6 related cytokines [59–61]. ORM has been revealed to have effects on immunoregulation, such as decreasing the rolling, adhesion, and migration of neutrophils [62–64]. In the ORM family, ORM1 is the unique member that could be considered as an acute-phase protein. ORM1 regulates the inflammation by contributing both anti- and pro-inflammatory signals to cytokine-mediated feedback mechanisms activated by the acute-phase response [65]. What’s more, ORM can also enhance the functional integrity of the blood-brain barrier (BBB) [66]. The study showed that decreasing the ORM1 expression may be a possible mechanism for the aggravation of BBB damage [67], while the disruption of BBB may underlie the occurrence of seizures and epilepsy [68, 69]. This study indicated that ORM1 may play important role in epilepsy.

ELANE encodes neutrophil elastase (NE), which belongs to the family of serine proteases. It has been reported that the mutations in the gene usually cause Cyclic neutropenia (CyN) and severe congenital neutropenia (SCN) [70, 71]. As NE is involved in immune responses and widely regarded as a regulatory factor in degenerative and inflammatory diseases through proteolysis of collagen-IV and elastin [72, 73], ELANE may be also associated with epilepsy.

The QPCT gene encodes glutaminyl cyclase (QC). QC is an enzyme responsible for catalyzing the posttranslational modification of N-terminal glutamate to pyroglutamate in many neuroendocrine peptides, which renders the protein more susceptible to neurotoxic [74]. Amyloid-β (Aβ) deposits have been found to be a characteristic neuropathological feature of Alzheimer’s disease (AD) [75]. N-terminally modified Aβ, pyroglutamate-amyloid-β (pE3-Aβ), is a major component of Aβ deposits specific to human AD [76, 77]. pE3-Aβ is processed by QC and/or its isoenzymes (isoQC), formed by cyclization of truncated Aβ species, rapidly aggregates and initiates other Aβ aggregates [78–80]. The formation of large amounts of pE3-Aβ has been shown to be QC-dependent. Reducing QC-dependent post-translational pE3-Aβ formation rate can in turn reduce the number of neurotoxic Aβ species [81]. As it is reported that in the pathophysiology of epilepsy Aβ may play an important role [82–84], QPCT may also serve as a novel strategy for the treatment of epilepsy.

FCAR encodes FcαRI or CD89, which is expressed on cells of the myeloid lineage [85] and is a bifunctional inhibitory/activating receptor for the Fc region of Immunoglobulin A (IgA) [86]. On one hand, FcαRI plays an anti-inflammatory role when binding to monomeric IgA and inducing inhibitory ITAMi signaling. On the other hand, when cross-linking IgA immune complexes, FcαRI mediates pro-inflammatory function, activating immune cells and leading to the elimination of pathogens [87, 88]. Neutrophilic activation is beneficial for infection; however, overabundant IgA complexes can trigger severe tissue damage causing various autoimmune diseases. Achiron A et al. found that FCAR participates in the pathogenic pathways in MS [89], which indicated FCAR may be involved in the various inflammatory responses in CNS. Targeting FcαRI might serve as a novel therapeutic strategy for epilepsy.

CD3G and CD3D encode CD3γ and CD3δ, respectively. Both CD3γ and CD3δ are part of the T-cell receptor/CD3 complex (TCR/CD3 complex) [90], which is crucial for the development, activation, and differentiation of T cells [91]. The CD3D gene defect, which occurs early in life, leads to severe immune deficiency, making a person susceptible to infection [92]. However, the CD3G mutation results in a milder clinical phenotype that is primarily autoimmune [93–95]. The severe immune deficiency may cause intracranial infection, while autoimmunity in brain may lead to autoimmune encephalitis, both of which are correlated to epilepsy [96–99]. However, the role of CD3G and CD3D in CNS is still not clear.

CLEC12A is a C-type lectin receptor (CLR) and a Src homology region 2 domain-containing phosphatase 1 and 2 (SHP-1 and -2)-associated receptor, highly expressed on human dendritic cells (DCs). CLEC12A contains a single immunoreceptor tyrosine-based inhibitory motif (ITIM) in its cytoplasmic tail. In response to chemokine CCL2, ITIM can associate with SHP-1 and SHP-2, involved in inhibitory signaling as a key molecule to deliver immature DCs to the CNS across the BBB [100]. It has been reported that DCs can initiate autoimmune demyelination and inflammation in CNS by presenting antigen to autoreactive myelin-specific T cells [101], while in the CLEC12A-KO mice the reduction of DC infiltration and demyelination was observed [102]. Thus, CLEC12A may be a promising target to inhibit seizures in brain.

MOSPD2 (motile sperm domain-containing protein 2) is the surface protein predominantly expressed on cytoplasmic membrane of human monocytes. MOSPD2 is also found in neutrophils, but not in lymphocytes. It is revealed that MOSPD2 is critical in regulating the inflammatory monocyte and neutrophil migration without activating ligands [103]. The monocyte from blood can rise macrophage in CNS, which is dominant in demyelination [104]. More and more researches suggest that chronic demyelination in multiple sclerosis can induce seizure [105–107]. As it has been demonstrated silencing or neutralizing MOSPD2 not only reduced the proportion of inflammatory monocytes in the blood significantly but also inhibited monocyte migrating into CNS [108], MOSPD2 may be a potential target for the treatment of epilepsy.

ALDH3B1 encodes the protein that belongs to the ALDH3 protein family (Aldehyde Dehydrogenase Family 3) [109–111]. It has been shown that ALDH3B1 plays a critical role in the cellular defense against oxidative stress processes and aldehyde toxicity [112, 113]. Oxidative stress toxicity and lowered antioxidant defense are considered as contributing factors in the genesis and progression of epilepsy [114–116], while epileptic seizures, especially recurrent seizures may also increase oxidative stress, which will result in treatment resistance [117–120]. Oxidative stress can lead to the occurrence of lipid peroxidation (LPO) and resulting in plenty of aldehydes, such as 4-hydroxy-2-nonenal (4HNE) [121]. In the CNS, dopamine is metabolized to 3,4-dihydroxyphenylacetaldehyde (DOPAL), while both norepinephrine and epinephrine are metabolized to 3,4-dihydroxyphenylglycol aldehyde (DOPEGAL) [122]. Aldehydes, including DOPAL, DOPEGAL, and 4HNE, are neurotoxic and involving in Parkinson’s disease (PD) and AD [123, 124]. ALDH7A1, the other member of the ALDH protein family, has been demonstrated to be related to pyridoxine-dependent epilepsy [125–127]. Thus, it is indicated that ALDH3B1 may have a protective role in various brain diseases including epilepsy.

GPR97 belongs to the G protein-coupled receptors (GPCRs), the largest receptor superfamily broadly involved in the regulation of biological processes and various diseases, including CNS disorders, such as anxiety, depression, schizophrenia, epilepsy, Alzheimer’s disease, and Parkinson’s disease [128, 129]. GPR97, expressed in immune cells and lymphatic endothelial cells [130, 131], contributes to macrophage-associated inflammation [132]. GPR97 also regulates the development of B-cell and NF-κB activity [133], which plays a critical role in encephalitogenic T cell activation [134]. The study reveals that the loss of GPR97 results in the increase of constitutive expression and activation of NF-κB pathway components, in turn causing severe inflammation and demyelination in CNS [135]. Thus, modulation of GPR97 functions or its pathway may be a potential treatment of epilepsy.

PLAUR encodes urokinase-type plasminogen activator receptor (uPAR) [136], which is a glycoprotein linked to the cell membrane by a glycosylphosphatidylinositol anchor [137]. The uPAR is a key regulator in many processes involving in not only cell signaling, proliferation, differentiation, and migration, but also tissue remodeling [137–139]. uPAR plays an important role in the early and injured brain. The uPA-uPAR complex induces axonal growth and regeneration by stimulating neuronal migration and neuritogenesis via both proteolytic and nonproteolytic events [140–142]. The dysregulation of uPA/uPAR axis is involved in various CNS disorders [139]. In rats undergoing seizures, expression of uPAR is increased in interneurons [143], while deficiency of both uPA and uPAR in mice increases seizure susceptibility [143–145]. In this study, the present findings also revealed that PLAUR was correlated with epilepsy.

Summarily, using the profile dataset and bioinformatics analysis, 16 epilepsy-associated hub genes were identified (Table 3). However, there are some limitations of this study. The lack of experimental evidence is probably the biggest limitation. In addition, the mechanism of these 16 hub genes in epilepsy is still unclear. Hence, more research should be carried out to investigate the functional roles of these hub genes in epilepsy.

**Table 3:** Summary of functions of hub genes.

Hub genes	Functions
ADCY7	Modulate the release of presynaptic GABA
C3AR1	Critical regulator in neuronal tau pathogenesis and mediating CNS immune network
DEGS1	Play an important role in sphingolipid signaling pathway and sphingolipid metabolism
CXCL1	Recruit and activate neutrophils in inflammation
TOLLIP	Inhibitory modulator in inflammatory signaling
ORM1	Regulate the inflammation and enhance the functional integrity of BBB
ELANE	Regulatory factor in degenerative and inflammatory diseases
QPCT	Catalyze the posttranslational modification of N-terminal glutamate of proteins to pyroglutamate in many neuroendocrine peptides
FCAR	Bifunctional inhibitory/activating receptor for the Fc region of IgA
CD3G and CD3D	Crucial for the development, activation, and differentiation of T cells
CLEC12A	Deliver immature DCs to the CNS across the BBB
MOSPD2	Critical in regulating the inflammatory monocyte and neutrophil migration without activating ligands
ALDH3B1	Play a critical role in the cellular defense against oxidative stress processes and aldehyde toxicity
GPR97	Contribute to macrophage-associated inflammation; regulate the development of B-cell and NF-κB activity involving in encephalitogenic T cell activation
PLAUR	Key regulator involving in not only cell signaling, proliferation, differentiation, and migration, but also tissue remodeling

## 5 Conclusion

In conclusion, by using the integrated bioinformatics analysis for gene expression profiles in epilepsy, we identified 16 hub genes, including sex-specific genes. These hub genes were correlated with the pathogenesis and prognosis of epilepsy. This study may contribute to further insight into epilepsy, by digging out the potential diagnostic and prognostic biomarkers, as well as therapeutic targets. Nevertheless, in the future, more research (in vivo and in vitro experiments) should be carried out to validate the functional roles of these genes in epilepsy.

## Supporting information

S1 Data (XLSX)
